# Timing of surgery for chronic subdural hematoma in patients with mild to moderate symptoms: a retrospective cohort study

**DOI:** 10.1007/s00701-025-06552-1

**Published:** 2025-05-19

**Authors:** M. Foppen, K.M. Slot, W.P. Vandertop, D. Verbaan

**Affiliations:** 1 Department of Neurosurgery, Amsterdam University Medical Center, University of Amsterdam, Meibergdreef 9, Amsterdam, Netherlands; 2 Amsterdam Neuroscience, Neurovascular Disorders, Amsterdam, The Netherlands

**Keywords:** Humans, Subdural, Hematoma, Chronic, Retrospective studies, Reoperation, Time Factors

## Abstract

**Background:**

Burr hole drainage is the mainstay of treatment for chronic subdural hematoma (cSDH). However, the impact of the interval between diagnosis and surgery on clinical outcome is unknown. This study investigates whether surgical timing affects outcome in patients with mild to moderate symptoms who do not require immediate surgery.

**Methods:**

We performed a single center, retrospective cohort study of 330 surgically treated cSDH patients with a Markwalder Grading Scale score of 1–2, at the Amsterdam UMC, between 2012 and 2022. The interval between diagnosis and surgery was measured in hours and dichotomized (surgery within vs. after 24 h). To account for potential confounding by hematoma mass effect, patients were stratified based on midline shift (greater than 10 mm vs < 10 mm). Primary outcomes included reoperation rate, complication rate, 30-day mortality, length of hospital stay and discharge destination. Univariable and multivariable regression analyses were performed for each stratum.

**Results:**

The mean age of the cohort was 73 years, and 241 (73%) were male. The median time to surgery was 25 h (IQR 15–54). Among the 330 patients, 157 (48%) underwent surgery within 24 h after diagnosis. Patients who received early surgery (< 24 h) had a significantly higher proportion of midline shift > 10 mm compared to those undergoing later surgery (56% vs. 34%, p < 0.001). The use of anticoagulant or antiplatelet therapy did not differ between groups (47% vs 54%, p = 0.27). No significant association was found between surgical timing and any primary outcome across all strata.

**Conclusion:**

In patients with cSDH presenting with mild to moderately symptoms, the timing of surgery did not affect clinical outcome, particularly as delayed surgery did not result in poorer outcomes. These findings suggest that postponing surgery to daytime hours may be safe in this subgroup. Validation in prospective studies, ideally incorporating functional outcomes, is nevertheless required to confirm these results and guide clinical practice.

**Supplementary Information:**

The online version contains supplementary material available at 10.1007/s00701-025-06552-1.

## Introduction

Chronic subdural hematoma (cSDH) is one of the most common neurological disorders encountered in neurosurgical practice [4, 7, 16, 19]. The standard treatment for symptomatic cSDH is surgical evacuation, typically through burr hole craniostomy.

Given the often gradual onset of symptoms, emergency surgery is rarely required. Logistical issues, such as interhospital transport from facilities without neurosurgical services, or the use of anticoagulant or antiplatelet medication, may contribute to delays between diagnosis and surgery [1, 3].

Despite the clinical relevance, studies examining the impact of the timing of surgery on patient outcome are limited [9, 14, 20, 22]. Consequently, evidence-based guidelines on the optimal timing of surgery are lacking, leading to significant variability in treatment practices across hospitals and countries [17]. Additionally, the influence of clinical condition and imaging parameters, such as midline shift or hematoma size, on the timing of surgery has been understudied. Also, current studies have not specifically concentrated on patients in whom the dilemma of surgical timing is prevalent. This includes individuals with mild to moderate symptoms, where their clinical condition may permit a waiting period. In contrast, patients experiencing severe symptoms, such as a significantly reduced level of consciousness, do not have the option of waiting and are instead immediately operated on to alleviate their condition. This further contributes to gaps in the existing literature.

This study aims to address this knowledge gap by evaluating whether the interval between diagnosis and surgery affects outcome in cSDH patients. Specifically, we focus on patients with a Markwalder Grading Scale (MGS) score of 1 and 2, assessing the association between surgical timing and key outcomes.

## Methods

**Fig. 1:**
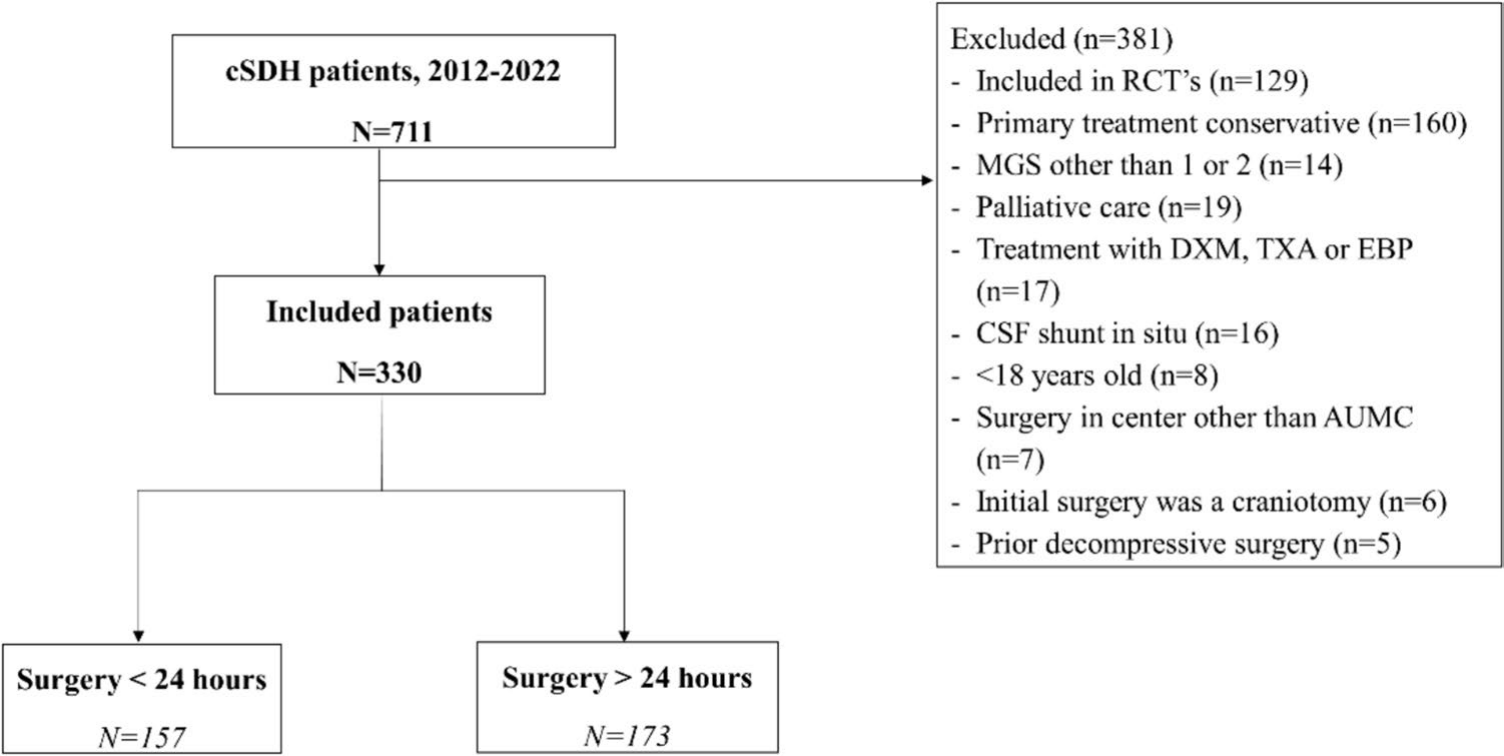
Flowchart of patient selection

We performed a single-center, retrospective cohort study of all 711 patients who were identified from a retrospective registry with a cSDH between 2012 and 2022 in the Amsterdam University Medical Center, a tertiary academic hospital in the Amsterdam Metropolitan area with a total population of approximately 2.5 million people. Patients were excluded if they met one of the following exclusion criteria: 1) a Markwalder Grading Scale score other than 1 or 2; 2) < 18 years old; 3) if the initial treatment strategy was conservative therapy (wait-and-watch); 4) decompressive cranial surgery performed within one year prior to diagnosis; 5) treatment with dexamethasone, tranexamic acid, epidural blood patch or palliative care; 6) cerebrospinal fluid shunt in situ at time of diagnosis; 7) surgical technique other than burr hole craniostomy (e.g. craniotomy) and drainage; 8) participation in a randomized controlled trial (TORCH-study or ELIMINATE-study [10, 11]; 9) surgery in a center other than Amsterdam UMC (see Fig. 1). Formal approval for this study was waived off by the local ethics committee and a waiver for informed consent was obtained (waiver number: 2024.0350).

### Treatment

The surgical procedure was uniform during the entire study period. All patients received burr hole craniostomy combined with drainage. The procedure was performed under general anesthesia in the operating theatre and pre-operatively, two grams of IV Cefazolin was administered. The hematoma was evacuated using one or two burr hole and the subdural cavity was rinsed with saline irrigation fluid at room or body temperature. The standard procedure involved placing a drain with passive suction, which was removed 24 h post-operatively. In our facility the interval between diagnosis and surgery was not standardized but general rules of thumb apply. Patients with life threatening symptoms or rapidly progressive neurological decline, received surgery immediately (directly or within eight hours). For patients who did not require surgical evacuation within eight hours, surgery was performed as soon as possible, but could be delayed due to logistical or facility-related issues (e.g. interhospital transport, admission delays owing to bed capacity constraints in our neurosurgical unit). If neurological decline occurred at any moment during the waiting period, surgery was advanced. For patients using anticoagulant or antiplatelet medication, surgery was usually performed after normalization of coagulation (antiplatelet therapy, 5–7 days; direct oral anticoagulants (DOACs), 48 h; vitamin K antagonists (VKA), after normalization of the INR ratio). If a patient’s clinical status did not allow for such a waiting period, DOACs and VKAs were reversed or thrombocytes were administered to be able to perform urgent surgery.

### Outcomes

Outcomes were 30-day mortality, reoperation and complication rate, length of hospital stay (LOS) and discharge location. Thirty-day mortality was noted as'yes'if death occurred within 30 days after diagnosis of the cSDH. For patients with a unilateral hematoma, reoperation was defined as ‘ipsilateral surgery for cSDH due to aggravation of symptoms or radiological progression of the hematoma during follow-up’. For patients with a bilateral hematoma, it was considered reoperation if repeated evacuation of the cSDH was required whether for one side or both [12]. The following complications were noted: all new post-operative intracranial hemorrhage and ischemic cerebrovascular events (confirmed by imaging), infections (post-operative wound infections, empyema, meningitis, pneumonia) which were culture-confirmed or if antibiotic therapy was started, wound leakage, seizures (if antiseizure medication was started), thrombo-embolic events and delirium (clinical diagnosis). Length of stay (LOS) was determined by calculating the difference between the cSDH admission date and discharge date from the neurological or neurosurgical unit. If patients were admitted more than once, or at the neurological or neurosurgical wards of other hospitals, the additional admitted days were also used to compute LOS. Patients were discharged to home, back to the referral hospital, a rehabilitation facility or nursing home.

### Data collection

Demographical (age, gender, hospital of diagnosis (neurosurgical center or referral hospital)), clinical (medical history, use of antiplatelet or anticoagulant therapy and clinical symptoms, occurrence of neurological deterioration while awaiting surgical intervention), radiological (hematoma location, type, basal cistern compression, size, volume and midline shift) and surgical parameters (drain type) were collected from the patients’ medical files. Hematomas were categorized as either mixed or homogeneous. The homogeneous type was further classified based on Hounsfeld Units (HU) into hypodense (HU < 25), isodense (HU 25–35) and hyperdense (HU > 35) hematomas (see supplementary Figs. 1 and 2) [8]. The presence of basal cistern compression was assessed according to the Rotterdam CT-score criteria for traumatic brain injury (normal basal cisterns, compressed or absent) [13]. The interval between diagnosis of the cSDH (moment of CT-scan) and surgery, was determined in hours by calculating the difference between their respective times and dates. Thirty-day mortality, complications, reoperations, length of stay and discharge location were also retrieved from the medical files. Duration of follow-up was derived by calculating the difference between the date of diagnosis and date of last follow-up visit.

### Statistical analysis

The normality of continuous variables was assessed with the Shapiro–Wilk test, considering a value > 0.9 as normally distributed. For normally distributed variables a mean and standard deviation (SD) were calculated and for not normally distributed variables a median and interquartile range (IQR) were calculated. Dichotomous variables were reported as numbers (%). According to the duration of the interval between diagnosis and surgery, two groups were established: 1) surgery within 24 h, 2) surgery 24 h or more after diagnosis. The degree of mass effect caused by the hematoma may impact surgical timing, as neurosurgeons tend to perform surgery more rapidly in cases with severe midline shift, independent of clinical status. Because of these considerations, we performed an additional analysis in stratified data, based on the amount of midline shift: patients with 1) less than 10 mm midline shift and 2) 10 or more than 10 mm midline shift. To describe the baseline differences between both groups before stratification, the appropriate parametric and non-parametric tests were used (Mann–Whitney U test, Fisher’s exact test, Chi-squared test and independent samples t-test). In this group, additional modelling for outcomes was not performed. In the strata, baseline differences between the groups according to timing of surgery were also described using appropriate parametric and non-parametric tests (Mann–Whitney U test, Fisher’s exact test, Chi-squared test and independent samples t-test). Next, univariate analysis using logistic and linear multivariable regression models of the groups'baseline characteristics was performed for each stratum. The outcomes reoperation, complication and 30-day mortality rates, length of hospital stay and discharge location per stratum were analyzed. Variables with p < 0.05 in univariate analyses were included into the corresponding multivariable regression model for that stratum. Finally, a subgroup analysis was performed to determine the effect of daytime versus after-hours surgery. Surgical procedures performed between 10:00 p.m. and 8:00 a.m. were considered after-hours operations. All patients were included and categorized as operated during daytime vs. after-hours. Univariate and multivariable regression analyses were conducted to evaluate the impact of the time of day at which surgery was performed on the study outcomes. All statistical analyses were performed with IBM SPSS statistics, version 28.0 and R version 4.3.2 (R Foundation for Statistical Computing, Vienna, Austria; http://www.R-project.org/) [18].

## Results

**Fig. 2:**
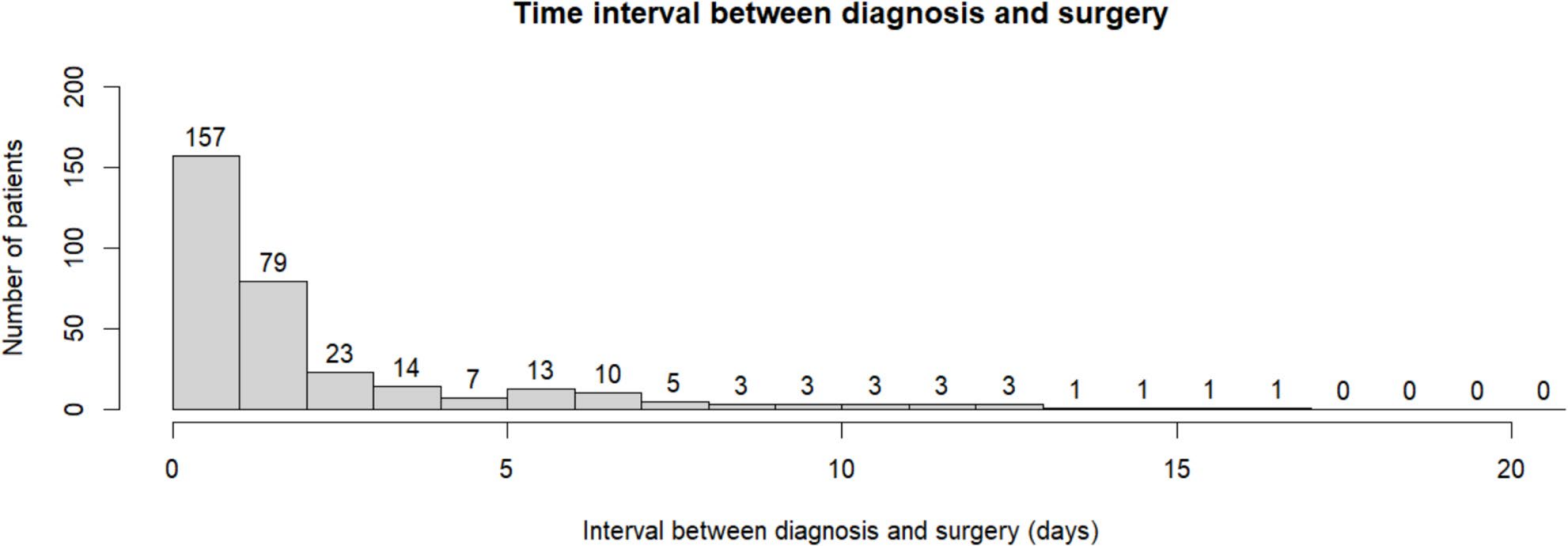
Time until surgery was longer than 20 days in three patients

The mean age of all included patients was 73 years (12 SD) and 241 (73%) were male. The cSDH was diagnosed in a referral hospital in 273 (83%) patients and the median time of follow-up was 62 days (IQR 48–83) (see Table 1 for all baseline characteristics). One hundred fifty-seven (47%) patients received surgery within 24 h after diagnosis. The median interval between diagnosis and surgery was 25 h (IQR 15–54, range 1 h—38 days) (see Fig. 2). The amount of midline shift (> 10 mm, 56% vs. 34%) and hematoma volume 130 ml vs. 149 ml) were significantly different between patients receiving surgery before or after 24 h. At the time of diagnosis, compression of the basal cisterns was also significantly more common in the group that underwent early surgical intervention (26% vs. 9%). Use of anticoagulant or antiplatelet therapy was not significantly different across both groups (47% vs 54%, *p*= 0.27).

**Table 1:** Baseline characteristics of 330 patients with cSDH

Variable	Total (n = 330)	Within 24 h (n = 157)	After 24 h (n = 173)	p-value
Age, mean (SD)	73 (12)	72 (12)	74 (11)	0.105^*a*^
Male, *n* (%)	241 (73)	114 (73)	46 (27)	0.969^*c*^
CSDH diagnosed at referral center, *n* (%)	273 (83)	122 (78)	151 (87)	0.031^*c*^
Arrhythmia, *n* (%)	79 (24)	37 (24)	42 (24)	0.983^*c*^
Cerebrovascular accident, *n* (%)	56 (17)	25 (16)	26 (15)	0.943^*c*^
Ischemic heart disease, *n* (%)	51 (16)	25 (16)	26 (15)	0.943^*c*^
DVT or PE, *n* (%)	14 (4)	7 (5)	7 (4)	1.000^*b*^
COPD, *n* (%)	31 (9)	19 (12)	12 (7)	0.156^*b*^
Diabetes, *n* (%)	94 (29)	39 (25)	55 (32)	0.202^*c*^
Alcoholism in history, *n* (%)	23 (7)	10 (6)	13 (8)	0.849^*c*^
AC or AP, *n* (%)	167 (51)	74 (47)	93 (54)	0.275^*c*^
Anticoagulation therapy, *n* (%)	88 (27)	43 (27)	45 (26)	
Antiplatelet therapy, *n* (%)	87 (27)	34 (22)	53 (31)	
Headache, *n* (%)	153 (46)	78 (50)	75 (44)	0.269^*c*^
Motor deficit, *n* (%)^8^	202 (65)	97 (66)	105 (65)	0.808^c^*
Pronation, drift or fall of arm/leg	58 (30)	27 (29)	31 (18)	
MRC 4	104 (53)	42 (45)	62 (36)	
MRC 3	19 (10)	14 (15)	5 (3)	
MRC 2	8 (4)	6 (7)	2 (1)	
MRC 1	2 (1)	1 (1)	1 (1)	
MRC 0	5 (3)	3 (3)	2 (1)	
Markwalder Grading Scale, *n* (%)				0.081^*c*^
1	76 (23)	29 (19)	47 (27)	
2	254 (77)	128 (82)	126 (73)	
Hematoma type^*39^				
Mixed, *n* (%)	180 (62)	92 (63)	88 (62)	0.440^c^
Hyperdense, *n* (%)	16 (5)	5 (3)	11 (8)	
Isodense, *n* (%)	28 (10)	14 (10)	14 (10)	
Hypodense, *n* (%)	65 (22)	35 (24)	30 (21)	
Basal cistern compression, *n* (%)^*¥*^	57 (17)	41 (26)	16 (9)	< 0.001^c^
Bilateral cSDH’s, *n* (%)	115 (35)	47 (30)	68 (39)	0.095^*c*^
Midline shift in mm (SD)	8 (5)	10 (5)	7 (5)	< 0.001^*a*^
Midline shift > 10 mm (%)	145 (44)	87 (56)	58 (34)	< 0.001^*c*^
Hematoma diameter in mm (SD)^**^	22 (7)	22 (8)	22 (7)	0.604^*a*^
Hematoma volume in ml (SD)^**^	133 (48)	130 (45)	149 (60)	0.014^a^
Drain type^4^				0.189^c^
Subdural	218 (67)	112 (71)	106 (61)	
Subgaleal/subperiosteal	84 (26)	36 (23)	48 (28)	
No drain	24 (7)	8 (5)	16 (9)	

^*a*^Independent samples t-test, ^*b*^Fisher-exact test, ^*c*^Chi-squared test.. DVT, deep venous thrombosis; PE, pulmonary embolism; COPD, chronic obstructive pulmonary disease; AC, anticoagulant therapy; AP, antiplatelet therapy; GCS, Glasgow Coma Scale; MRC, Medical Research Council scale

*In 39 patients the hematoma type could not be determined. In fifteen cases, the diagnosis was made based on MRI findings, and in twenty-four cases, due to the presence of bilateral hematomas with differing characteristics on each side

** In patients with a bilateral cSDH, the hematoma with the maximum diameter or volume was used for analysis

¥ Among the 57 patients presenting with some degree of basal cistern compression, complete obliteration of the cisterns was not observed in any case

Table 2 summarizes the outcomes of the total study population. The general reoperation rate was 10% (n = 34) and total complication rate in all patients was 18% (n = 59). Most common were seizures and delirium (20 patients, 6%). The median length of hospital stay was six days (IQR 3–12). One hundred fifty-one (46%) patients could be discharged to their home. One hundred fifty-three (46%) patients were transferred to another hospital and 24 (7%) were discharged to a rehabilitation facility. Two patients died in hospital; one due to an acute postoperative subdural hematoma and one as a result of multiple recurrences complicated by acute intracranial hemorrhage. Nine (3%) patients died within 30 days after diagnosis. Fifty-nine (18%) patients experienced neurological decline while awaiting surgery. A decrease in Glasgow Coma Scale score was the most frequently occurring form of neurological decline (Table 1, Supplementary).

**Table 2:** Outcomes in all patients

Variable	
Number of cases	330
30-day mortality, *n* (%)^21^	9 (3)
Reoperation, *n* (%)	34 (10)
Complications, *n* (%)	59 (18)
Length of hospital stay, days (IQR)	6 (3–12)
Discharge location	
Home	151 (46)
Other	179 (54)
Transfer to other hospital	153 (46)
Rehabilitation facility	24 (7)
All complications	
Post-operative acute subdural hemorrhage, *n* (%)	8 (2.4)
Other intra-cranial hemorrhage, *n* (%)	4 (1.2)
Ischemic cerebrovascular event, *n* (%)	2 (0.6)
Wound infection or leakage, *n* (%)	11 (3.3)
Empyema/meningitis, *n* (%)	9 (2.7)
Seizure, *n* (%)	20 (6.1)
Thrombo-embolic event, *n* (%)	3 (0.9)
Delirium, *n* (%)	20 (6.1)
Pneumonia, *n* (%)	11 (3.3)

Amount of total complications is higher than 59 as some patients experienced more than one complication. ^*b*^Fisher-exact test, ^*c*^Chi-squared test, ^*d*^Mann-Whitney U-test. In 21 patients 30-day mortality was unknown as their follow-up was shorter than 30 days

One hundred eighty four (56%) patients had less than 10 mm midline shift (see Table 3 for baseline characteristics). In this group, significantly more subdural drains were placed in patients treated within 24 hours. In the regression analyses, timing of surgery was not related to any of the outcomes (Table 4).

**Table 3:** Baseline characteristics of 184 patients with cSDH and <10 mm midline shift

*Variable*	*Within 24 hours (n=69)*	*After 24 hours (n=115)*	*p*-value
Age, mean (SD)	71 (13)	75 (10)	0.078^a^
Male, *n* (%)	46 (67)	85 (74)	0.377^c^
CSDH diagnosed at referral center, *n* (%)	55 (80)	100 (87)	0.273^c^
Arrhythmia, *n* (%)	15 (22)	30 (26)	0.626^c^
Cerebrovascular accident, *n* (%)	9 (13)	19 (17)	0.672^c^
Ischemic heart disease, *n* (%)	9 (13)	19 (17)	0.672^c^
DVT or PE, *n* (%)	5 (7)	3 (3)	0.153^b^
COPD, *n* (%)	12 (17)	9 (8)	0.083^c^
Diabetes, *n* (%)	18 (26)	33 (29)	0.832^c^
Alcoholism in history, *n* (%)	1 (1)	9 (8)	0.133^c^
AC or AP, *n* (%)	29 (42)	63 (55)	0.128^c^
Headache, *n* (%)	37 (54)	49 (43)	0.213^c^
Motor deficit, *n* (%)	44 (66)	68 (63)	0.841^c^
Pronation, drift or fall of arm/leg	13 (30)	19 (28)	
MRC 4	21 (48)	41 (60)	
MRC 3	6 (14)	3 (4)	
MRC 2	3 (7)	0 (0)	
MRC 1	0 (0)	1 (1)	
MRC 0	1 (2)	2 (3)	
Markwalder Grading Scale, *n* (%)			0.275^c^
1	14 (20)	33 (29)	
2	55 (80)	82 (71)	
Hematoma type^27^			0.595^c^
Mixed, *n* (%)	43 (68)	56 (60)	
Hyperdense, *n* (%)	2 (3)	7 (7)	
Isodense, *n* (%)	6 (10)	10 (11)	
Hypodense, *n* (%)	12 (19)	21 (22)	
Basal cistern compression, *n* (%)	11 (16)	8 (7)	0.100^c^
Bilateral cSDH’s, *n* (%)	34 (49)	58 (50)	1.000^c^
Midline shift in mm (SD)	5 (3)	4 (3)	0.056^a^
Hematoma diameter in mm (SD)	21 (7)	21 (7)	0.484^a^
Hematoma volume in ml (SD)	124 (44)	115 (44)	0.177^a^
Drain type			0.035^c^
Subdural drain	54 (78)	68 (60)	
Subgaleal/subperiosteal drain	10 (15)	34 (30)	
No drain	5 (7)	11 (10)	

^a^Independent samples t-test, ^b^Fisher-exact test, ^c^Chi-squared test. DVT, deep venous thrombosis; PE, pulmonary embolism; COPD, chronic obstructive pulmonary disease; AC, anticoagulant therapy; AP, antiplatelet therapy; GCS, Glasgow Coma Scale; MRC, Medical Research Council scale.

**Table 4:** Outcome in 184 patients with <10 mm midline shift

**Variable**	Reoperation^¥^		Complications^¥^		>30-day mortality^¥^		LOS^*^		Discharge to home^¥^	
	aOR**	**OR **	aOR**	**OR **	aOR**	**OR **	aβ **	**β **	aOR**	**OR **
Surgery <24 hours	Reference	Reference	Reference	Reference	Reference	Reference	Reference	Reference	Reference	Reference
Surgery after 24 hours (95% CI)	0.72 (0.22–1.59)	0.63 (0.24–1.65)	0.89 (0.41–1.90)	0.98 (0.47–2.07)	1.03 (0.17–6.40)	0.96 (0.16–5.88)	1.09 (−2.24–4.44)	2.18 (−1.42–5.78)	1.02 (0.56–1.88)	0.94 (0.52–1.72)

^­^**Drain type was significantly different in univariate analyses. Therefore an adjusted odds ratio (aOR) and adjusted Beta (aβ) were calculated, accounting for drain type. ^¥^Analyzed using logistic regression, *analyzed using linear regression. CI, confidence interval; LOS, length of hospital stay.

Midline shift was greater than 10 mm in 154 (44%) patients (Table 5). Despite stratification, amount of midline shift remained significantly different between patients treated within, or after 24 h. None of the outcomes were related to timing of surgery in the multivariable regression analyses (Table 6).

**Table 5:** Baseline characteristics of 145 patients with cSDH and >10 mm midline shift

*Variable*	*Within 24 hours (n=87)*	*After 24 hours (n=58)*	*p-value*
Age, mean (SD)	73 (12)	74 (13)	0.638^a^
Male, *n* (%)	67 (77)	42 (72)	0.666^c^
CSDH diagnosed at referral center, *n* (%)	66 (76)	51 (88)	0.112^c^
Arrhythmia, *n* (%)	22 (25)	12 (21)	0.660^c^
Cerebrovascular accident, *n* (%)	16 (18)	7 (12)	0.430^c^
Ischemic heart disease, *n* (%)	16 (18)	7 (12)	0.430^c^
DVT or PE, *n* (%)	2 (2)	4 (7)	0.218^b^
COPD, *n* (%)	7 (8)	3 (5)	0.738^c^
Diabetes, *n* (%)	21 (24)	22 (38)	0.111^c^
Alcoholism in history, *n* (%)	9 (10)	4 (7)	0.678^c^
AC or AP, *n* (%)	45 (52)	30 (52)	1.000^c^
Headache, *n* (%)	40 (47)	26 (45)	0.927^c^
Motor deficit, *n* (%)	53 (67)	37 (66)	1.000^c^
Pronation, drift or fall of arm/leg	14 (26)	12 (32)	
MRC 4	21 (40)	21 (57)	
MRC 3	8 (15)	2 (5)	
MRC 2	3 (6)	2 (5)	
MRC 1	1 (2)	0 (0)	
MRC 0	2 (4)	0 (0)	
Markwalder Grading Scale, n (%)			0.421^c^
1	75 (86)	48 (83)	
2	12 (14)	10 (17)	
Hematoma type^12^			0.434^c^
Mixed, *n* (%)	49 (58)	32 (65)	
Hyperdense, *n* (%)	3 (4)	4 (8)	
Isodense, *n* (%)	9 (10)	4 (8)	
Hypodense, *n* (%)	23 (27)	9 (18)	
Basal cistern compression, *n* (%)	30 (35)	8 (14)	0.011^c^
Bilateral cSDH’s, *n* (%)	12 (14)	10 (17)	0.741^c^
Midline shift in mm (SD)	14 (3)	12 (2)	<0.001^a^
Hematoma diameter in mm (SD)	24 (8)	23 (6)	0.460^a^
Hematoma volume in ml (SD)	155 (47)	146 (44)	0.254^a^
Drain type			0.191^c^
Subdural drain	58 (67)	38 (67)	
Subgaleal/subperiosteal drain	26 (30)	14 (25)	
No drain	2 (2)	5 (9)	

^a^Independent samples t-test, ^b^Fisher-exact test, ^c^Chi-squared test. DVT, deep venous thrombosis; PE, pulmonary embolism; COPD, chronic obstructive pulmonary disease; AC, anticoagulant therapy; AP, antiplatelet therapy; GCS, Glasgow Coma Scale; MRC, Medical Research Council scale.

**Table 6:** Outcome in 145 patients with >10 mm midline shift

Variable	Reoperation^¥^		Complications^¥^		30-day mortality^¥^		LOS^*^		Discharge to home^¥^	
	aOR**	OR	aOR**	OR	aOR**	OR	aβ **	β	aOR**	OR
Surgery <24 hours	Reference	Reference	Reference	Reference	Reference	Reference	Reference	Reference	Reference	Reference
Surgery after 24 hours (95% CI)	0.74 (0.22–2.46)	0.73 (0.23–2.25)	0.50 (0.17–1.44)	0.51 (0.19–1.40)	0 (0-inf)	0 (0-inf)	0.54 (−1.69–2.77)	0.36 (−1.81–2.52)	1.06(0.52–2.17)	0.94 (0.61–1.46)

**The amount of midline shift and basal cistern compression yes/no remained significantly different in univariate analyses. Therefore an adjusted odds ratio (aOR) and adjusted Beta (aβ) were calculated, accounting for amount of midline shift and presence of basal cistern compression. ^¥^Analyzed using logistic regression, *analyzed using linear regression. CI, confidence interval; LOS, length of hospital stay.

### Subgroup analysis

Surgery was performed during after-hours in 51 patients. These patients had significantly more often a MGS 2 (90% vs. 74%, p = 0.024) and a larger hematoma volume (149 ml vs. 130 ml, p = 0.014), compared to surgeries performed during daytime (Table 2, Supplementary). After correction for these variables in the multivariable analysis, after-hours surgery was not associated with any outcome (Table 3, Supplementary).

## Discussion

This single center, retrospective cohort study of patients with a cSDH, who presented with mild to moderate symptoms and did not require immediate surgery, demonstrates that timing of surgery was not associated with any of the outcome parameters evaluated.

Timing of surgery for cSDH is at the discretion of the neurosurgeon on call who generally makes a decision, based on clinical status, radiological features and, not in the least, on logistical practicalities. Surprisingly, only two studies, have investigated this topic [20, 22]. The first study is a prospective cohort study performed in the United Kingdom, including symptomatic uni- and bilateral cSDH patients (n = 656), who all received burr hole craniostomy [20]. The median time to surgery was one day (range 0–44 days). This study showed that the interval between diagnosis and surgery was not associated with clinical outcome (mRS at discharge, mortality, complications and reoperation rate). The second study is a retrospective cohort study (n = 179) in Sweden, which only included patients with a unilateral cSDH, and the surgical procedure was a mini craniotomy [22]. The median time to surgery was approximately three days, notably longer than the median wait of one day in our study. The outcomes were similar to ours, as there was no significant relationship between timing of surgery and Glasgow Outcome Scale, mortality, LOS and discharge to home. However, reoperation rate was significantly higher when the interval between diagnosis and surgery was reduced [22].

It is reasonable to assume that patients who receive surgery sooner, are in a more severe clinical condition than patients who are operated on at a later stage. However, we only included patients with mild to moderate symptoms (MGS 1 or 2), and the MGS was evenly distributed between patients receiving surgery within or after 24 h, suggesting that factors other than neurological compromise influenced the timing. The two prior studies did not make a distinction based on clinical severity and included generally ‘worse patients’. In the Swedish study almost 5% of all cases had a Glasgow Coma Scale (GCS) less than 12 (which implies a MGS of 3 or 4), and in the British study almost 30% had a pre-operative GCS of less than 12. The clinical implication of the results of our study, would be that there is no need for after-hours surgery in cSDH patients with mild to moderate symptoms since timing does not affect outcome. This allows for surgeries to be scheduled during daytime. In academic hospitals in the Netherlands, such as our institution, surgeries for patients with mild to moderate symptoms, are mostly scheduled in a semi-urgent, non-elective, setting. In the context of our countrywide classification system for emergency surgeries, this would classify as ‘surgery within 24 h after enrollment on the emergency surgery list’ [5]. Therefore, a cut-off period of 24 h was used, as it mirrors the dilemma faced by neurosurgeons who must weigh the safety of deferring surgery against the potential for neurological decline, or proceed with after-hours intervention. Daytime procedures should be prioritized when feasible, as after-hours interventions in general, are associated with worse outcomes. Although our subgroup analysis did not reveal poorer outcomes for surgeries performed during after-hours, a systematic review including almost three million cases (all surgical disciplines), demonstrated that nighttime surgery was associated with a higher risk of mortality (OR 1.16; 95% CI, 1.06–1.2), compared to daytime procedures [6, 21]. The results of two recent other studies, support the notion of deferring surgery until daytime if possible. They investigated the outcomes of daytime vs. after-hours surgery, particularly in patients with cSDH, and did not demonstrate beneficial outcomes of after-hours surgery [9, 14]. The first study is a British cohort study (*n* = 263) [9]. In this study, no significant differences were found with regard to outcomes (recurrence, complications, mortality), between both groups. However, the results of both groups were only compared, without adjusting for possible confounders such as clinical status, radiological features and time interval between diagnosis and surgery. The second study is a post-hoc analysis of a Finnish RCT (n = 589, 17% after-hours surgery) [14], in which the outcomes (mRS, mortality, and reoperation rates at six months) did not show significant differences between daytime and after-hours surgeries in either group. In this study, correction was performed for variables associated with surgical timing (such as pre-operative clinical status, midline shift and hematoma diameter). However, half of the patients did not receive peri-operative subdural irrigation. This is standard practice (80–100%) in most parts of the world, and the primary RCT showed that the use subdural irrigation decreases recurrence rate significantly [2, 15].

### Limitations and future perspectives

A limitation of our study is the retrospective nature, which makes the results susceptible to confounding and bias. The relatively long study period, ensured that drain type was not standardized and could be either a subdural or subgaleal/subperiosteal (SPG) drain. However, in most cases a subdural drain was inserted. Furthermore, body-temperature irrigation fluid was integrated in the standard operating procedure after the study period, in 2023. Before that period, either room or body temperature was used, but this information could not be extracted from operative notes. In order to completely eliminate bias, a randomized controlled trial must be performed, comparing early vs. late surgery. However, purposely delaying surgeries is unlikely to be implemented in daily clinical practice. Therefore, the added value of such an RCT is low. Observational studies like these, reflecting real-world scenarios more accurately, are therefore highly valuable. Another drawback of our study is the inability to investigate how surgical outcomes might vary if performed at times other than within, or beyond 24 h. For example, if surgery was postponed for five to seven days, in patients using antiplatelet medication. A closer examination of the day-by-day effects of postponing surgery after the first 24 h was not possible, as most surgeries were performed within 48 h. Another limitation of our study may be the exclusion of brain atrophy from our analysis, as our focus was on volumetric parameters such as midline shift, hematoma volume, and compression of the basal cisterns. Accurately evaluating brain atrophy on CT scans is challenging, especially in the presence of large subdural hematomas. This assessment typically requires a baseline scan obtained before hematoma formation, which was not available in the majority of patients. Finally, patient-reported outcomes, such as function (modified Rankin Scale) and Quality of Life (the SF-36), were not included because they were not systematically reported in the patients’ medical files. Future prospective studies, incorporating these outcomes, are required to determine the effect of surgical timing on these domains.

## Conclusion

In this retrospective cohort study including mild to moderately affected cSDH patients who do not require immediate surgery, the timing of surgery did not affect clinical outcome, particularly as delayed surgery did not result in poorer outcomes. Prospective studies incorporating functional outcomes are required to determine whether postponement of surgery to daytime hours can be done safely.

## Supplementary Information

Below is the link to the electronic supplementary material.

Supplementary file1 (DOCX 5.36 MB)

## Data Availability

No datasets were generated or analysed during the current study.
