# Supplementary material for: Timing of surgery for chronic subdural hematoma in patients with mild to moderate symptoms: a retrospective cohort study
Source: Acta Neurochir (Wien). 2025 May 19;167(1):147. doi: 10.1007/s00701-025-06552-1 (PMC12089212; doi:10.1007/s00701-025-06552-1)
Supplement: Supplementary file 1 — Supplementary file1 (DOCX 5.36 MB) [file 701_2025_6552_MOESM1_ESM.docx]

**Figure 1**


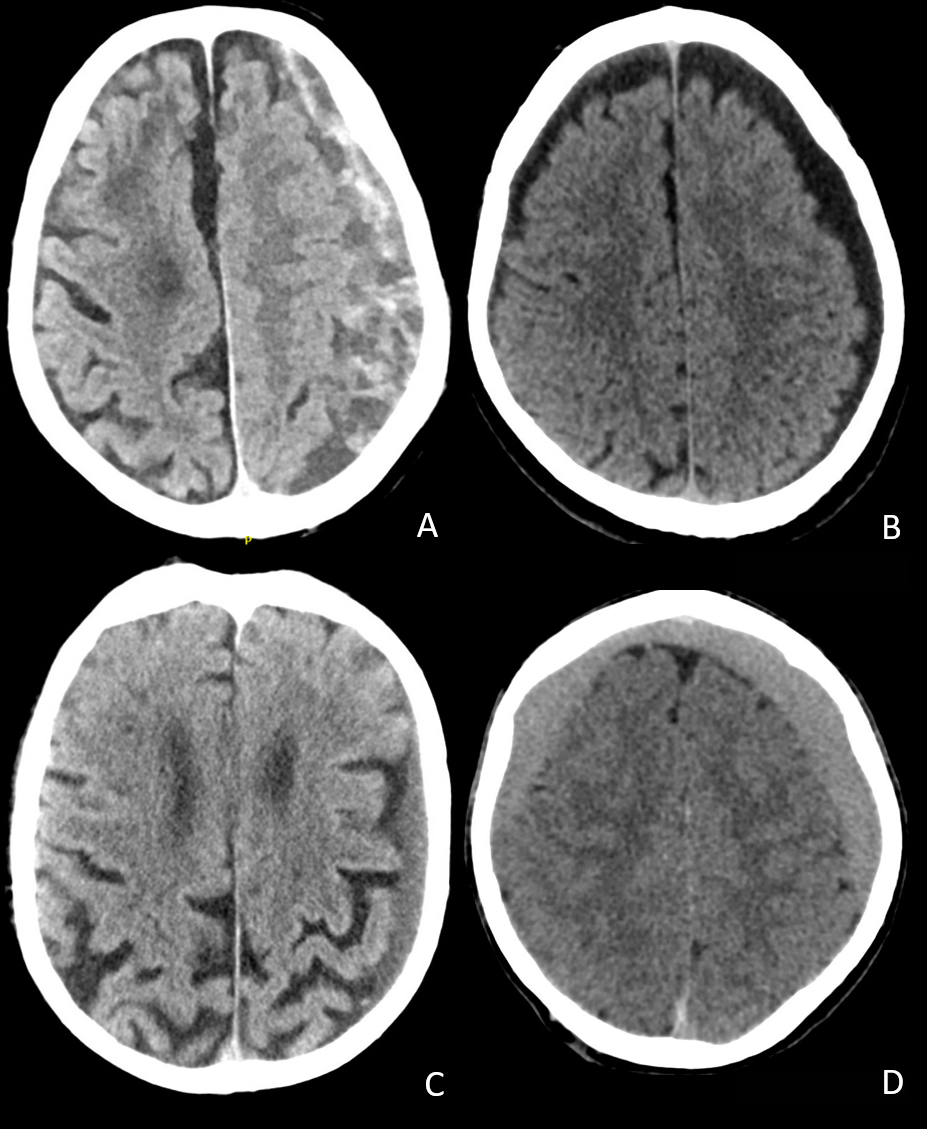


Classification of hematoma type. A mixed type hematoma, B homogeneous hypodense hematoma, C homgeneous isodense hematoma, D homogeneous hyperdense hematoma

**Figure 2**


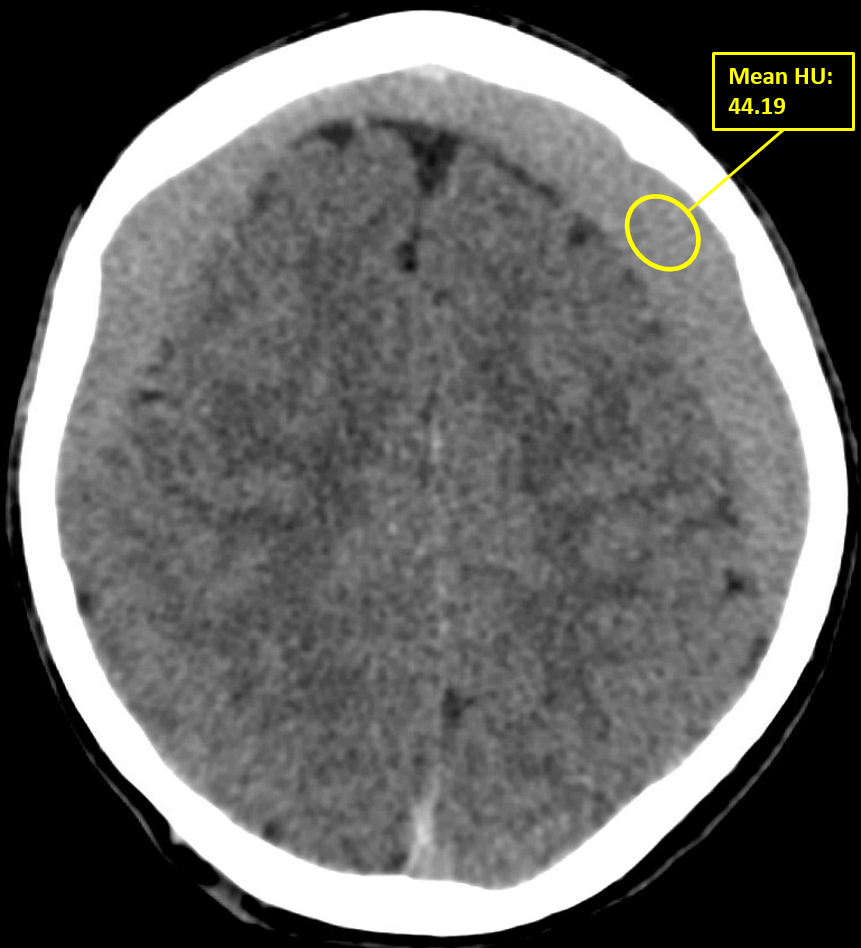


Method of assessing average amount of HU for homogeneous hematomas.

| **Table 1. Neurological decline awaiting surgery** | |
| --- | --- |
| **Aggravated symptoms** | **Number of patients (%)** |
| GCS | 26 (44) |
| Multiple symptoms | 16 (27) |
| Motor deficit | 10 (17) |
| Language disorder | 3 (5) |
| Epileptic seizures | 3 (5) |
| Gait disorder | 1 (2) |
| *Twenty-six patients experienced a decrease in Glasgow Coma Scale (GCS) score awaiting surgery. In sixteen patients multiple symptoms progressed.* | |

| **Table 2. Characteristics daytime vs. after-hours surgery** | | | |
| --- | --- | --- | --- |
| *Variable* | *Daytime surgery (n=279)* | *After-hours surgery (n=51)* | *p-value* |
| Age, mean (SD) | 73 (12) | 73 (10) | 0.795*^a^* |
| Male, n (%) | 205 (73) | 36 (70) | 0.798^c^ |
| CSDH diagnosed at referral center, n (%) | 232 (83) | 41 (80) | 0.781^c^ |
| Arrhythmia, n (%) | 64 (23) | 15 (29) | 0.414^c^ |
| Cerebrovascular accident, n (%) | 39 (14) | 12 (24) | 0.127^c^ |
| Ischemic heart disease, n (%) | 39 (14) | 12 (24) | 0.127^c^ |
| DVT or PE, n (%) | 12 (4) | 2 (4) | 1.000^b^ |
| COPD, n (%) | 25 (9) | 6 (12) | 0.711^c^ |
| Diabetes, n (%) | 82 (29) | 12 (24) | 0.494^c^ |
| Alcoholism in history, n (%) | 19 (7) | 4 (8) | 0.764^b^ |
| AC or AP, n (%) | 135 (48) | 32 (63) | 0.083^c^ |
| Headache, n (%) | 125 (45) | 28 (55) | 0.266^c^ |
| Motor deficit, n (%) | 174 (65) | 28 (65) | 1.000^c^ |
| Pronation, drift or fall of arm/leg | 52 (31) | 6 (21) |  |
| MRC 4 | 88 (53) | 16 (57) |  |
| MRC 3 | 15 (9) | 4 (14) |  |
| MRC 2 | 7 (4) | 1 (4) |  |
| MRC 1 | 2 (1) | 0 (0) |  |
| MRC 0 | 4 (2) | 1 (4) |  |
| Markwalder Grading Scale, n (%) |  |  | 0.024^c^ |
| 1 | 71 (26) | 5 (10) |  |
| 2 | 208 (74) | 46 (90) |  |
| Hematoma type^29^ |  |  | 0.441^c^ |
| Mixed, n (%) | 153 (62) | 27 (61) |  |
| Hyperdense, n (%) | 15 (6) | 1 (2) |  |
| Isodense, n (%) | 26 (11) | 3 (7) |  |
| Hypodense, n (%) | 52 (21) | 13 (30) |  |
| Basal cistern compression, n (%) | 47 (17) | 10 (20) | 0.806^c^ |
| Bilateral cSDH’s, n (%) | 97 (35) | 18 (35) | 1.000^c^ |
| Midline shift in mm (SD) | 8 (5) | 9 (5) | 0.147*^a^* |
| Hematoma diameter in mm (SD) | 22 (7) | 23 (10) | 0.206*^a^* |
| Hematoma volume in ml (SD) | 130 (45) | 149 (60) | 0.014*^a^* |
| Drain type |  |  | 0.536^c^ |
| Subdural drain | 187 (68) | 31 (62) |  |
| Subgaleal/subperiosteal drain | 68 (25) | 16 (32) |  |
| No drain | 21 (8) | 3 (6) |  |
| *^a^Independent samples t-test, ^b^Fisher-exact test, ^c^Chi-squared test. DVT, deep venous thrombosis; PE, pulmonary embolism; COPD, chronic obstructive pulmonary disease; AC, anticoagulant therapy; AP, antiplatelet therapy; GCS, Glasgow Coma Scale; MRC, Medical Research Council scale.* | | | |

| **Table 3. Outcomes after-hours surgery** | | | | | |
| --- | --- | --- | --- | --- | --- |
|  | **Reoperation^¥^** | **Complications^¥^** | **30-day mortality^¥^** | **LOS^*^** | **Discharge to home^¥^** |
| **Variable** | **aOR**** | **aOR**** | **aOR**** | **aβ**** | **aOR**** |
| Daytime surgery | Reference | Reference | Reference | Reference | Reference |
| After hours surgery | 0.66 (0.22-2.04) | 1.00 (0.45-2.24) | 2.70 (0.63-11.39) | 0.99 (-0.44-2.43) | 0.75 (0.39-1.42) |
| *******Markwalder Grading Scale (MGS) and hematoma volume were significantly different in the univariate analysis. Therefore, an adjusted odds ratio (aOR) and adjusted Beta (aβ) were calculated, accounting for MGS and volume. ^¥^Analyzed using logistic regression, *analyzed using linear regression. CI, confidence interval; LOS, length of hospital stay.* | | | | | |
